# The interface between the national tuberculosis control programme and district hospitals in Cameroon: missed opportunities for strengthening the local health system –a multiple case study

**DOI:** 10.1186/1471-2458-13-265

**Published:** 2013-03-22

**Authors:** Basile Keugoung, Jean Macq, Anne Buve, Jean Meli, Bart Criel

**Affiliations:** 1 Ministry of Public Health, Cameroon; Research, Education, and Health Development Group (GARES-Falaise), Dschang, Cameroun; 2 Institut de Recherche Santé et Société, Université Catholique de Louvain, Brussels, Belgium; 3 Public Health Department, Institute of Tropical Medicine, Nationalstraat 155, Antwerp, Belgium; 4 Faculty of Medicine and Biomedical Sciences, University of Yaounde I, Yaounde, Cameroon

**Keywords:** Case study, Cameroon, District hospital, Programme, Sub-Saharan Africa, Tuberculosis

## Abstract

**Background:**

Tuberculosis remains a major public health problem in sub-Saharan Africa. District hospitals (DHs) play a central role in district-based health systems, and their relation with vertical programmes is very important. Studies on the impact of vertical programmes on DHs are rare. This study aims to fill this gap. Its purpose is to analyse the interaction between the National Tuberculosis Control Programme (NTCP) and DHs in Cameroon, especially its effects on the human resources, routine health information system (HIS) and technical capacity at the hospital level.

**Methods:**

We used a multiple case study methodology. From the Adamaoua Region, we selected two DHs, one public and one faith-based. We collected qualitative and quantitative data through document reviews, semi-structured interviews with district and regional staff, and observations in the two DHs.

**Results:**

The NTCP trained and supervised staff, designed and provided tuberculosis data collection and reporting tools, and provided anti-tuberculosis drugs, reagents and microscopes to DHs. However, these interventions were limited to the hospital units designated as Tuberculosis Diagnostic and Treatment Centres and to staff dedicated to tuberculosis control activities. The NTCP installed a parallel HIS that bypassed the District Health Services. The DH that performs well in terms of general hospital care and that is well managed was successful in tuberculosis control. Based on the available resources, the two hospitals adapt the organisation of tuberculosis control to their settings. The management teams in charge of the District Health Services are not involved in tuberculosis control. In our study, we identified several opportunities to strengthen the local health system that have been missed by the NTCP and the health system managers.

**Conclusion:**

Well-managed DHs perform better in terms of tuberculosis control than DHs that are not well managed. The analysis of the effects of the NTCP on the human resources, HIS and technical capacity of DHs indicates that the NTCP *supports,* rather than *strengthens,* the local health system. Moreover, there is potential for this support to be enhanced. Positive synergies between the NTCP and district health systems can be achieved if opportunities to strengthen the district health system are seized. The question remains, however, of *why* managers do not take advantage of the opportunities to strengthen the health system.

## Background

It is not likely that the health-related Millennium Development Goals will be met by 2015 in many low-income countries, especially in sub-Saharan Africa [1,2]. In most of these countries, health care delivery does not cover the basic health needs of the population, and the quality of health care is poor [3].

Vertical programmes have been created to organise activities and control major diseases, such as HIV/AIDS, tuberculosis, and malaria. These programmes receive technical and financial support from donors and international organisations. However, despite the progress that has been made, many of these diseases remain a major public health threat in most low-income countries [4-6].

Currently, most studies on the interface between vertical programmes and general health services have focused on the effects of programmes on health systems in general [7-9]. These studies have been conducted at the community and first-line health service levels [9,10]. They have also concentrated on the outputs produced by health services [11-13]. Studies on the impact of vertical programmes on district hospitals (DHs) are rare despite the central role played by DHs in local health systems [14]. This study aims to fill this gap. Consequently, having a clear picture of the results of the interaction between DHs and vertical programmes may improve our understanding of the challenges that district health systems face in resource-limited settings. Such understanding can better enable the delivery of quality health care.

Indeed, tuberculosis control policies and strategies, and the relation between tuberculosis control programmes and general health services have evolved over time. The delivery of tuberculosis care has alternately been organised either through a vertical approach using specialised tuberculosis health services or via a more integrated approach in general multi-purpose health services. Currently, WHO recommends the ‘Direct Observed Short Term Strategy’ (DOTS), and a proper balance between integration and specialisation, and between decentralisation and centralisation [15]. Therefore, researches are needed to assess tuberculosis control programmes implementation in various settings so as to progressively identify adequate strategies that are built from the strengths and the weaknesses of the current approaches [16].

We hypothesise that the results of the interaction between vertical programmes and general health services depend on the characteristics of the programme and the performance of the general health service. In this study, we focused on the recipient health system by investigating the effects of the National Tuberculosis Control Programme (NTCP) on DHs in Cameroon.

In Cameroon, tuberculosis is diagnosed and treated at health facilities (mainly hospitals) that are accredited by the NTCP. Thus, this context represents an interesting setting for analysing the interface between the NTCP and DHs. The objectives of this study are to analyse the results achieved by the DHs in terms of tuberculosis control; to analyse whether and how the NTCP affects human resources, health information systems (HIS) and the technical capacity of DHs in Cameroon; and, finally, to investigate whether the effects of the NTCP on DHs vary between hospitals.

## Methods

### Study design

We used a multiple case study methodology. A case study design was found to be the appropriate method for this investigation because our research questions aimed to explore and explain whether, and especially how, the NTCP affects DHs. Moreover, we sought to compare these effects between different DHs. We were able to investigate this complex issue using a limited sample of cases [17-20].

We carried out two case studies in two rural DHs in the same region, a public hospital, and a private-not-for-profit (pnfp) Lutheran hospital. These hospitals were selected on the basis of the following criteria: i) they should be the only hospitals in their respective districts so that their outputs can be understood relative to the district population; ii) they should have an operational Tuberculosis Diagnosis and Treatment Centre (TDTC), as health facilities with a TDTC interact more with the NTCP; iii) they should have a similar geographical context; iv) the TDTC should be of relatively recent creation (no more than 10 years) so that the hospital staff can recall changes, but it should be old enough (at least five years) to facilitate the study of these changes; and v) they should have different institutional identities and governance cultures to allow the comparison of the NTCP effects between the two settings. Two DHs located in the Adamaoua Region, met the criteria and were selected, and a TDTC was created for both hospitals in 2003. For ethical reasons, the two DHs and the health districts in which they are located will be labelled District Hospital A (DHA) in case of the pnfp hospital, and District Hospital B (DHB) in case of the public hospital.

We chose DHs in Adamaoua region because it was the last region in Cameroon where the TDTC approach was introduced. We hypothesise that the experience gained in other regions may have been used to design the most effective possible strategy for tuberculosis control in this region.

In case study research, the distinction between ‘*holistic’* and ‘*embedded’* designs is made [20]. We opted for an ‘embedded’ multiple-case design, meaning that more than one unit of analysis is used for each case. The units of analysis in our research were the performance of the DH, and effects of the NTCP on the human resources, HIS and technical capacity of the DH. We collected both qualitative and quantitative data in this study. Quantitative data were analysed using Microsoft Excel 2007. Medians, means and ratios were calculated where appropriate. We compared the two hospitals for each unit of analysis.

### Study settings

The Cameroon health system has been structured in the following three levels since 1995: central, regional and district. In 2010, there were 10 health regions, 178 health districts, and more than 3000 health facilities. At the district level, the District Health Service (DHS) is run by the district management team, which is responsible for organising, supervising and coordinating all health activities. Most districts cover rural populations and have only one hospital while in urban health districts, more than one hospital can be found. All public DHs have roughly similar organization with a managerial team appointed by the Ministry of Public Health [21].

The NTCP was created in 1995, started its field operations in 1996 in one region and gradually expanded to cover the entire country in 2003. A total of 16 TDTCs existed in 1996, and the NTCP operationalised 216 TDTCs in 2009, 87% of which were located in hospitals. The objectives of the NTCP are to detect at least 70% of smear-positive pulmonary tuberculosis (SPPT) cases and to cure at least 85% of these patients [22].

In 2010, the Adamaoua Region had 8 health districts of which six are rural: Bankim, Banyo, Djohong, Meiganga, Tibati, and Tignere and two are urban: Ngaoundere I and Ngaoundere II [23]. In this region, which had a total population of 1,015,622 inhabitants, there were 134 formal health facilities: one public regional hospital, 7 DHs (5 public and 2 pnfp) and 126 first-line health services (91 public, 31 pnfp, and 4 private-for-profit). Since 2003, only 9 (7%) health facilities had a TDTC (1 regional hospital, 7 DHs and 1 first-line public health service). Before 2003, in the Adamaoua Region, each health facility acquired reagents and drugs for tuberculosis care from its proper funds. Moreover, data on tuberculosis were not standardized and drugs were not free of charge [22].

In 2010, the health district A had 152,167 inhabitants (population density: 11 inhabitants/km^2^), 15 ‘health areas’ with 25 first-line health services (19 public, 4 pnfp and 2 private-for-profit). The health district B had 95,267 inhabitants (density: 9 inhabitants/km^2^), 12 ‘health areas’ and 16 first-line health services (13 public and 3 pnfp). The two hospitals became DHs in the late 90s following the adoption of the district-based health system organisation in Cameroon [24].

### Conceptual frameworks and data collection

To analyse the performance of the two DHs, we used the framework developed by Van Lerberghe et al. [25], who assessed sub-Saharan DHs on the basis of the following three different dimensions: spatial, managerial and technical (see Table 1). First, in the spatial dimension, the DH is considered to be an element of a system, which, in this study, is the district health system. Second, the DH is analysed as an organisation that should be adequately managed to achieve a good performance. Third, the DH is a technical structure that delivers health care.

**Table 1:** Framework for collecting data on DH performance

**Dimension**	**Data collected**	**Source of information**
Spatial dimension	Attraction zone	Interviews
	Peripheral structures	Document review
	Supervision of health centres	Interviews
	Coordination meetings	Interviews
	Referral system	Interviews
Managerial dimension	Resource generation	Document review, interviews, observation
	Resource management	Interviews
	Management quality	Interviews
Technical dimension	Staff (technical and support staff)	Document review
	Amenities for patients	Observation
	Technical equipment	
	Tuberculosis care indicators	Document review
	General health care indicators	Document review

For our study of the effects of the NTCP on the human resources, HIS and technical capacity of DHs, we used three different conceptual frameworks.

First, we analysed the NTCP’s effects on human resources using a framework (see Table 2) developed by Diallo et al. [26]. The framework proposes indicators for evaluating human resources for health in relation to two of the four core functions of a health system (health service provision and resource generation) [27].

**Table 2:** Framework for assessing the effects of the NTCP on the human resources of DHs

**Domain**	**Data collected**	**Source of information**
**Health service provision**		
Distribution of hospital staff in general wards and in the TDTC	Number and type of personnel recruited by the NTCP	Interviews
Identification of the TDTC nurse	Criteria for recruiting or identifying the TDTC staff	Interviews
Internal migration from general health care to tuberculosis activities	Number and type of staff dedicated to tuberculosis activities (date)	Interviews
Implementation of tuberculosis control activities	Type and number of staff involved in tuberculosis care	Interviews Observation
	Role of each staff member	
Staff incentives from the NTCP	Salaries provided by the NTCP	Document review Interviews
	Type and amount of incentives related to tuberculosis activities	
	Provider of the incentives	
**Human resource generation**		
Recruitment of staff for tuberculosis control	Date and reason for change	Interviews
Training and supervision of staff	Type and date of training by the NTCP	Document review Interviews
	Number and type of personnel trained or supervised	
	Content of the training	
	Competencies acquired for general health care	
Supervision	Frequency	Document review Interviews Observation
	Supervisors	
	Supervisees	
	Subject of supervision	
	Process of supervision	
Acquisition of skills for health care delivery	For tuberculosis activities	Interviews
	For general health care	

Second, we used the framework developed by Aqil et al. [28] to study the effects of the NTCP on HIS (see Table 3). This framework is called the ‘Performance of Routine Information System Management framework’ and implies that the performance of routine HIS is affected by HIS processes as well as by technical, organisational and behavioural factors [29,30].

**Table 3:** Framework for assessing the effects of the NTCP on the routine HIS of DHs

**Factors**	**Domain**	**Data collected**	**Sources of information**
**Technical factors**	Reporting system	Type of new tools introduced by the NTCP and their use	Interviews
		Availability of tuberculosis and routine information tools	Observation
	Designer of reporting forms	Designer of the routine information system	Interviews
		Changes in the design of routine information after TDTC creation	
	Complexity of the reporting forms	Complexity of the tuberculosis and routine information tools	Interviews
	Procedures	Rules for tuberculosis and routine data collection,analysis and transmission	Document review
		Changes in routine HIS procedures following the creation of the TDTC	Interviews
**Organisational factors**	Information distribution	Type of reports sent by the hospital before and after the creation of the TDTC	Interviews
		Services receiving hospital reports	
	Interest devoted to reporting	Motivation of the TDTC staff for reporting	Interviews
		Motivation of the ward staff members for reporting	
	Quarterly HIS supervision	Staff members supervised in the use of thetuberculosis HIS (frequency)	Interviews Observation
		Staff members supervised in the use of the routine HIS (frequency)	
	Training	Training received on HIS management: trainees and date	Interviews
**Behavioural factors**	Level of knowledge of content of HIS forms	Staff members involved in monthly routine reporting	Interviews
		Knowledge of the content of HIS tools by hospital staff	
	Skills	Skills in data collection, processing and analysis	Interviews
	Motivation	Level of motivation	Interviews
**Processes**	Data collection	Data completeness in registers (tuberculosis and routine care data)	Observation
	Data processing	Availability of tuberculosis and routine reports(period) at the hospital level	Observation
	Data analysis	Type of analysis conducted on tuberculosis data and on routine data	Interviews Document review
	Data transmission	Availability of tuberculosis and routine reports (period)at the district and regional levels	Interviews Observation
	Data display andfeedback to nurses	Type of data displayed (for tuberculosis and routine care data)	Observation
	Data quality checking	Procedures of data checking and actors involved	Interviews

Finally, we analysed the effects of the NTCP on the technical capacity of DHs using a framework based on the main elements of ‘essential medical products and technologies’.

‘Essential medical products, vaccines and technologies’ is one of the 6 key components of a well-functioning health system, which are as follows: essential medical products, vaccines and technologies; leadership and governance; human resources; HIS; health financing; and health service delivery [31]. We investigated the effects of the NTCP on medications, technical and office equipment, logistics supplies, infrastructure rehabilitation and/or construction in the two hospitals. For each item, data were collected via interviews and through observation.

### Data collection

We collected data between August 2011 and February 2012 using the following three data collection techniques: document review, interviews and observation.

Document review

The documents reviewed were the hospital registers; the monthly, quarterly and annual hospital reports; and the annual district reports. At the regional level, we studied the general and tuberculosis-specific annual reports as well as the directives and guidelines produced by the NTCP.

Interviews

We conducted 35 semi-structured interviews using an interview guide. In total, 3 interviews were conducted at the regional level, 6 at the district level, 20 at the hospital level, and 6 at the first-line health service level. We used a purposive sampling of interviewees. Interviewees were selected on the basis of their responsibilities in the health system and their experience in offering tuberculosis care. At the district level, we selected the district medical officers, hospital directors, nurses in charge of a ward, nurses in charge of the TDTC, and head nurses at first-line health services (health centres). Starting from the initial selection, other participants were identified using a snow-ball strategy until we reached saturation. At the regional level, three health professionals associated with NTCP coordination were interviewed.

The interview guide was adapted after a preliminary analysis of each interview. Notes were taken during interviews and were used to complete the interview content. The interviews were conducted in French, lasted from 30 to 90 minutes and were audio-taped. A full transcription of all interviews was written using Microsoft Word 2007 software. We used NVivo 9 QSR International Pty Ltd software (Victoria, Australia) to analyse the interviews.

Observation

We observed the supervision of tuberculosis activities at the TDTC level –these supervision were done by the NTCP staff from the regional and central levels -, the staff meetings, the routine work of staff, and the flow of patients between and within hospital units. We made an inventory of the infrastructure, equipment, medications and staff available per unit.

### Ethical issues

We obtained ethical clearances from the Institutional Review Board of the Institute of Tropical Medicine, Antwerp, Belgium (N° 772), and the Cameroon National Ethics Committee (N° 113/CNE/SE/09 and N° 258/CNE/SE/2011). Administrative approvals were delivered by the Cameroon Ministry of Public Health (N° 631.9-10), the NTCP (N° 0925) and the regional health authorities (634/L/MSP/SP/DRSP/A/NGD). Each interviewee signed an informed consent form to participate after a full explanation of the research objectives was provided. Finally, in order to minimise the risk of revealing the identity of the interviewees, the pnfp DH was labelled DH A (DHA) and the public DH was labelled DH B (DHB).

## Results

The results are presented in two parts. In the first section, we analyse and compare the two hospitals, and in the second part, we present the effects of the NTCP on both hospitals.

### Performance of DHA and DHB

#### Spatial dimension

The DHA supervises, coordinates, and allocates resources to its network of 5 faith-based health centres. However, the hospital is not involved in these activities at public facilities. At DHB, DHS staff members and health centre nurses noted that the supervision of health centres was mainly conducted during mass immunization campaigns organized by the Expanded Programme of Immunization against diseases such poliomyelitis, measles or yellow fever’. Only one administrative hospital staff member was involved in these supervisions. Ten mass campaigns were organised in 2011.

The referral system is not well organised in either district, nor is it well organised in Cameroon in general [32]. In both health districts, health centre nurses revealed that no feedback is received from DHs regarding referred patients. In both hospitals, differentiation between referred and non-referred patients is not always provided in registers of outpatient consultations.

The median distances between the health centres and the DH are 58 and 86 km in A and B health district respectively. As is the case with other faith-based hospitals in Cameroon [33,34], DHS staff explained that DHA has a good reputation that attracts patients living even beyond the district boundaries.

#### Managerial dimension

In DHA, the Hospital Management Board meets weekly to discuss all hospital issues (see Table 4). There is strong leadership. Staff are managed at the hospital level in terms of salaries, promotions, appointments to a health centre in their network, and sanctions. For example, a query for a written explanation was given to a nurse who arrived two hours late to work, and cases were repeatedly reported of staff being dismissed due to poor performance. In terms of resource generation, the DHA is quite successful. A manager stated that ‘*when the tuberculosis ward deteriorated, we succeed in obtaining 11 million FCFA*^*a*^ (approximately 25,000 US$) *for its renovation from a European Organisation’.* Regarding the organisational culture, the hospital is highly influenced by faith-based values.

**Table 4:** Managerial and technical dimensions of DHs A and B performance

	**Characteristics**	**District hospital A**	**District hospital B**
Governing bodies	Committee	Regional faith-based coordination committee of the Health	Hospital management committee
		Hospital Management Board	
Leadership	Leadership strength	Strong	Weak
		Shared	Centralised at the directorate level
Resources generation	User fees collected	222.49 million FCFA 86% of revenues managed by the hospital in 2010	37.9 million FCFA, 77% of revenues managed by the hospital in 2010
	Subsidies from the Ministry of Health	Irregular funding (35.4 million FCFA in 2010) used on the basis of hospital needs	Regular lump funding every six months used on the basis of directives from the central directorate (progressively decreased from 15.4 million FCFA in 2001 to 10.84 million FCFA in 2010)
		No wages paid to staff	Wages to technical staff
	Search for external funding from donors	Pro-active	Low
		Regular external support in terms of technical expertise, equipment, drugs, infrastructure rehabilitation and construction from foreign organisations	Little support from local associations in 2010 (beds and mattresses)
Management	Scope of management practices	Related to faith-based values	Administrative procedures
	Financial resources	Based on hospital needs and strategic plans	Based on guidelines from the Ministries of Health and of Finances, and are bureaucratic
	Human resources	Decentralised management by hospital committees	Centralised management
	Feedback to staff	Openly discussed at weekly meetings	Rare, with some aspects withheld
	Maintenance	Well-equipped support services (e.g., woodwork, electricity and plumbing)	Scarce support service
	Support and administrative staff	22	9
Human resources	Number of staff	Technical staff: 41	Technical staff: 23
	Medical doctors	2 (2003–2006); 3 (2007–2010)	1 (2003–2006); 3(2007–2010)
	Inhabitants per medical doctor	50,872 inhabitants	23,817 inhabitants
Equipment	Number of beds	157	49
	Technical equipment	Good	Poor
		Radiograph, 2 echographs, Mammograph, cardiotopograph, 2 well-equipped surgical theatres, electronic sphygmomanometers in each ward, oxygen	A small surgical theatre with little equipment
	Amenities for patients	High-quality ward	Not available
		Tap water and electricity permanently available	Tap water only available in the morning, frequent electricity cut-offs

While, in DHB, the Hospital Management Committee meets quarterly to mainly discuss issues relating to the management of user fees. Staff tend to refer problems to the hospital director, who seems to have little authority. The management decisions are made centrally at the Ministries of Public Health, Finances and Civil Service. A nurse stated that ‘*when people go on leave, they return sometimes two to three weeks after the end of their leave’.* The only administrative measure that can be used against such staff in DHB is to issue a query for a written explanation (in French: *demande d’explication)*. The hospital relies mostly on subsidies from the Ministry of Public Health for its running costs and wages. The organisational culture is of a more bureaucratic nature, and is based on the use of administrative procedures [21].

#### Technical dimension

Resources

The capacity of the DHA (157 beds) is three times higher than that of DHB (49 beds). The DHA is technically well-equipped and has more amenities for patient comfort (see Table 4). There was a 30-beds ward for tuberculosis inpatients and a nurse only in charge of tuberculosis patients. Additionally, support services for administrative duties, hygiene and sanitation, and maintenance are well-equipped, while these services are scarce and poorly-equipped in DHB. In 2007, the DHB welcomed two medical doctors and 10 nurses from the Ministry of Public Health while in the health district A only public health centres received these additional staff.

General health care indicators

The number of outpatients in the DHA has progressively decreased from 143 patients per 1000 inhabitants in 2002 to 50 patients per 1000 inhabitants in 2010 (see Table 5). Interviewees explained that, due to the creation of many new public and private health centres, the number of primary cases received at the hospital level had progressively diminished, and currently, cases received are more severe –suggesting a more appropriate pattern of health services utilisation in the district. Indeed, the proportion of outpatients hospitalised increased from 15% in 2003 to 46% in 2010. Despite the poor registration of referred and counter referred patients, the referred patients registered represent 4% to 6% of outpatients. Admission rates were approximately 24 inpatients per 1000 inhabitants between 2002 and 2010.

In the DHB, the number of outpatients decreased from 32 to 27 outpatients per 1000 inhabitants between 2002 and 2006 and rose after 2006 to reach 52 outpatients per 1000 inhabitants in 2010. The referred patients among outpatients represent 1% to 4%. Interviewees highlighted that additional staff, as well as new laboratory equipment received in 2007 contributed to improve the capacity and the functionality of the hospital. Admission rates ranged between 8 and 16 inpatients per 1000 inhabitants, and had progressively increased since 2007.

Tuberculosis control at the DHA and DHB

**Figure 1:**
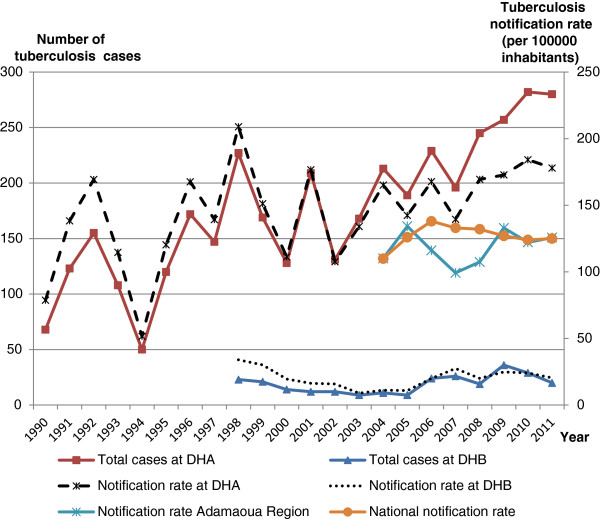
**Number of tuberculosis cases at DHA and DHB, and notification rates of all cases of tuberculosis at DHA, DHB, Adamaoua region and the national level At DHB, data on tuberculosis care between 1990 and 1997 were not available.** Also at regional level, data on tuberculosis were only available after 2003 when the DOTS strategy was introduced in the Region.

Data on tuberculosis control activities were available from tuberculosis registers from 1990 to 2011 and from 1998 to 2011 at the DHA and DHB respectively, while a synthesis of the Adamaoua regional tuberculosis data was only available since 2004. The tuberculosis notification rates of the DHA from 1998 to 2011 were 5 to 15 times higher than those of the DHB (see Figure 1). Between 2004 and 2011, the notification rates of the DHA were 1.1 to 1.6 times higher than those of the Adamaoua region that were at the same time 3.6 to 12.3 higher than the notification rates of the DHB. From 2009 to 2011, the Adamaoua notification rates were similar to the national notification rates.

The DHA achieved the NTCP objective of detecting at least 70% of SPPT cases per year despite the presence of a second TDTC in the district, whereas the DHB detected less than 50% of cases. However, some patients come from outside the district boundaries. One staff member of DHB explained that ‘*before 2006, the hospital was just like a health centre, and the laboratory could only perform the same exams as in health centres’*. The DHB did not have the necessary equipment –such as a radiograph, reagents for biopsy conservation - to improve the diagnosis of extra-pulmonary and smear negative pulmonary tuberculosis cases. This contrasts with DHA that was in a position to perform chest radiography and biopsies on suspected tuberculosis cases with negative sputum smears. Since 2003 –when data collection for tuberculosis care was standardized- SPPT cases ranged between 25% and 65% of all tuberculosis cases in DHA, and between 64% and 100% in DHB.

From 2005 to 2010, cure rates were between 51 and 84% at DHA, and between 61 and 78% at DHB.

**Table 5:** General health care indicators in the DHs A and B

**Hospital**	**Indicators**	**2002**	**2003**	**2004**	**2005**	**2006**	**2007**	**2008**	**2009**	**2010**
A	Outpatients received	16956	20177	13773	8852	11673	11168	10937	9177	7398
	Outpatients/1000 inhabitants per year	143	166	110	69	88	82	78	63	50
	Inpatients	NA	3014	3247	3140	3196	3308	3371	3268	3386
	Inpatients/1000 inhabitants/year	NA	25	25	24	24	24	24	23	23
B	Outpatients received	2463	2878	2113	2191	2271	2445	3183	3505	4986
	Outpatients/1000 inhabitants per year	32	37	26	27	27	28	35	38	52
	Inpatients	744	790	738	675	716	954	1147	1538	1539
	Inpatients/1000 inhabitants per year	10	10	9	8	8	11	13	17	16

NA: Not Available.

### Effects of the NTCP on DHs

In the second part of our results, we present the effects of the NTCP on the human resources (see Table 6), technical capacity (see Table 7) and HIS (see Table 8) of DHA and DHB. The interventions of the NTCP are first described, followed by the effects of the NTCP on the routine system. In addition, a review of the adaptive strategies used by hospital managers to ensure tuberculosis care, and of missed opportunities that were not seized to build synergies between the NTCP and the local health system is provided.

**Table 6:** Effects of NTCP on the level of human resources for health of the DHs A and B

**Health service provision**	**District hospital A**	**District hospital B**
Stock of personnel	No additional staff recruitment	No additional staff recruitment
Criteria for identifying the TDTC staff	Availability, seriousness, obligingness	Availability, seriousness, obligingness
Internal migration from general health care to TB activities	Since 2008, one assistant nurse dedicated to the TDTC	Partial migration: the TDTC nurse was head of a ward (surgery from 2003 to 2008 and medicine since 2009)
	One laboratory technician dedicated to sputum smear processing	
Labour force activity	Detection of suspect tuberculosis patients by consulting nurses Processing of sputum smears by a dedicated laboratory technician	Detection of suspect TB patients mainly by medical doctors
	Drugs dispensation, follow up of hospitalized tuberculosis patients and reporting by the TDTC nurse	Processing of sputum smears by all laboratory technicians
	Chest radiography by a specialized nurse	Drugs dispensation and reporting by head nurse of the medicine ward
**human resources generation**		
Earnings	No staff paid by the NTCP	No staff paid by the NTCP
Incentives	15000 FCFA given to the TDTC nurse per trimester since 2010	15000 FCFA given to the TDTC nurse per trimester since 2010
	Fees for sputum smear managed by the TDTC nurse	Fees for sputum smear included in hospital revenues
Productivity	No patient increase following the TDTC creation	No patient increase following the TDTC creation
Education and training	Competencies gained on counselling, treatment of respiratory tract infections, smear processing and reading of slides on microscope by trained staff	Competencies gained on counselling, treatment of respiratory tract infections, smear processing and reading of slides on microscope by trained staff
	Workshops organized on tuberculosis care for TDTC staff and hospital managers	Workshops organized on tuberculosis care for TDTC staff and hospital managers
	Quarterly supervision of the TDTC staff by the NTCP coordinators	Quarterly supervision of the TDTC staff by the NTCP coordinators

**Table 7:** Effects of the NTCP on the technical capacity of DHs A and B

**Frequency**	**Type of inputs**	**District hospital A**	**District hospital B**	**Observations**
Permanent allocation since 2003	Drugs	Anti-tuberculosis drugs for adults and children	Anti-tuberculosis drugs for adults only	Frequent out-of-stocks registered
	Reagents	Sulfuric acid, Methylene blue, Fuschin	Sulfuric acid, Methylene blue, Fuschin	Reagents used for sputum smear processing; used for other tests for non-TB patients
	Other laboratory materials	Slides and sputum collectors	Slides and sputum collectors	Equipment used for all patients
Sporadic allocation	Logistics	One motorcycle in 2006	No motorcycle	The motorcycle is used for other hospital outreach activities
	Equipments	Two electric microscopes in 2003 and 2007	Two electric microscopes 2003 and 2006	Equipment used for all patients
	Infrastructures Rehabilitation	No rehabilitation	Rehabilitation of a small building in 2006	The unit rehabilitated in 2006 at the DHB is out of use
	Finances	15000 FCFA quarterly allocated to each TDTC	15000 FCFA quarterly allocated to each TDTC	Office equipment insufficient in both TDTC
		Sputum smear fees collected and managed by the TDTC nurse		

**Table 8:** Effects of the NTCP on routine health information system at DHs A and B

**Technical factors**	**District hospital A**	**District hospital B**
Reporting system	Printed tuberculosis tools (registers, patient treatment card, 2 quarterly reporting forms) introduced by the NTCP in 2003	Printed tuberculosis tools (registers, patient treatment card, 2 quarterly reporting forms) introduced by the NTCP in 2003
	Printers registers for routine HIS	Registers manually designed for routine HIS
Designer of reporting forms	NTCP for the tuberculosis HIS	NTCP for the tuberculosis HIS
		Ministry of health for the routine reporting form
		Managers of the hospital for registers
	Central level of the church for registers and reports	
Software for HIS	No	No
	Computers acquired from hospital resources	Computers acquired from hospital resources
Recruitment of a HIS staff	No for tuberculosis HIS	No
	Yes, in 2008, but only in charge of routine reporting and paid from hospital revenues	
Skills of the HIS staff in using computer	No specific training on HIS management	No specific training on HIS management
Complexity of the reporting forms	Simple for tuberculosis tools but takes too much time	Simple for tuberculosis tools but takes too much time
	Filling routine registers is easy	Filling routine registers is easy
Procedures	Simple	Simple
**Organisational factors**		
Information distribution	Reports sent to the regional NTCP coordination since 2003 (completeness: 100%)	Reports sent to the regional NTCP coordination since 2003 (completeness: 100%)
	Routine reports sent to the district till 2006, but regularly to the Church hierarchy	Routine reports sent to the district in 2010
Interest devoted to reporting	Very high for the NTCP	Very high for the NTCP
	Low for routine reports	Low for routine reports
Supervision	Quarterly by the NTCP coordinators, all tuberculosis tools reviewed	Quarterly by the NTCP coordinators, all tuberculosis tools reviewed
	Rare for routine activities	Rare for routine activities
Training	No specific training on HIS	No specific training on HIS
Finances	No additional resources for HIS	No additional resources for HIS
Allocation of computer	Computers acquired from hospital resources	Computers acquired from hospital resources
Allocation of reporting forms and other materials	Tuberculosis reporting tools provided by the NTCP	Tuberculosis reporting tools provided by the NTCP
	Routine registers provided by the Church	
**Behavioural factors**		
Level of knowledge of content of HIS forms	Very good for tuberculosis HIS, low for staff working in ward	Very good for tuberculosis HIS, low for staff working in ward
Data quality checking skills	Good for the TDTC nurse	Good for the TDTC nurse
	Routine data rarely checked	Routine data checked by the Director
Competency in HIS tasks	Low	Low
Motivation	Very high for the TDTC staff	Very high for the TDTC staff
	Low for other staff	Low for other staff
Problem solving tasks	Only raw data transmitted	Only raw data transmitted
**Processes**		
Data collection	Data rigorously filled in tuberculosis registers	Data rigorously filled in tuberculosis registers
	Incomplete routine data collection	Incomplete routine data collection
Data processing	All quarterly tuberculosis reports done since 2003	All quarterly tuberculosis reports done since 2003
	Lot of missing routine reports	All routine monthly reports done since 1998
	Routine reports not done since 2006	
Data analysis	Little analysis	Little analysis
Data transmission	Completeness : 100% for tuberculosis reports	Completeness : 100%
	Routine information transmitted only to the faith-based hierarchy	Only the 2010 routine reports sent to the district level
Data display	No	No
Data quality checking	Yes for tuberculosis reports	Yes for tuberculosis reports
	No for routine reports	Rarely for routine reports
Feedback to ward nurses	No	No

### Effects of the NTCP on human resources

Health care provision

In the DHA, the nurse in charge of the tuberculosis ward was changed quarterly until 2006 (see Table 6). Following the NTCP instructions, one laboratory technician processed all sputum smears, and one assistant nurse was assigned the task of permanently taking care of tuberculosis patients. A hospital manager however argued that staff were regularly changed so that more nurses could master tuberculosis care and to prevent individual staff members from being overexposed to the tuberculosis bacilli. He/she added that this strategy contributed to maintaining the continuity of care. The TDTC nurse noted that because ‘*I am alone, I receive patients until the office closes, and I do not have time to trace defaulters’*. The defaulting rate of SPPT was 9% in DHA in 2010 and in 2011, but dropped in DHB from 17% in 2010 to 6% in 2011.

**Figure 2:**
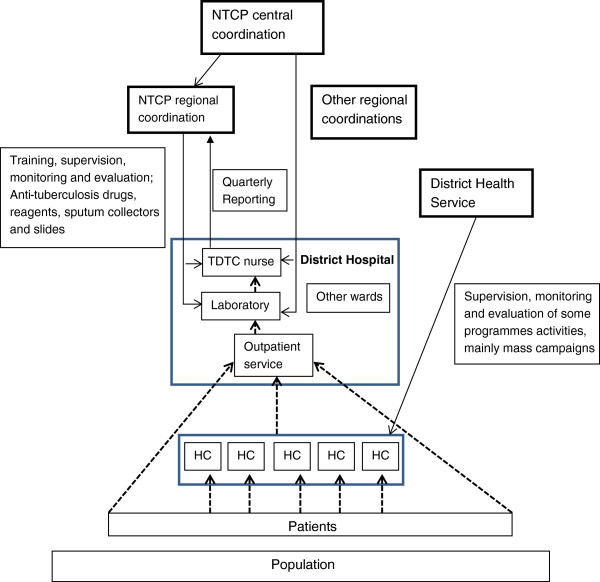
**Organisation of tuberculosis control activities in health districts A and B.** HC: Health Centre; TB: Tuberculosis. Patient flow health service activities.

In the DHB, the TDTC nurse was the head nurse of the surgery ward from 2003 to 2008 and has been the head of the medicine ward since 2009. All laboratory technicians processed sputum smears. The TDTC nurse noted that being the only person to deliver care to tuberculosis patients ‘*represents a problem for continuity of tuberculosis care; when I am absent or on leave, nobody is qualified to follow patients’.* He further added that ‘*this does not influence too much my work as head nurse of the medicine ward, as I follow only around 30 tuberculosis patients per year’.* A regional tuberculosis coordination staff member noted that with nurses specifically designated for tuberculosis control, following up on recommended measures for TDTC was easier. Regarding the organisation of tuberculosis care, suspected tuberculosis patients follow more or less the same flow as any other patient undergoing consultation for curative care in both hospitals. The consulting staff of both hospitals screens suspected tuberculosis cases among outpatients and sends them to the laboratory for a sputum smear examination. Additionally, at DHA, chest radiography is performed and interpreted by a specialised nurse. After the diagnosis of tuberculosis is made, the patients are referred to the TDTC nurse. The TDTC nurse delivers anti-tuberculosis drugs, fills in the tuberculosis data collection tools and gives quarterly reports. Health centre nurses are only passively used in tuberculosis control (see Figure 2). The two TDTC nurses were unable to identify interventions for tuberculosis control directed toward health centre staff. In both hospitals, the criteria mentioned by managers for selecting the TDTC nurses were availability, seriousness, and attention to their duties.

Human resource generation

The NTCP has not allocated additional staff to either hospital. Regarding competencies, the district medical officer, the director of the hospital, a nurse and a laboratory technician from each district were trained in clinical and laboratory diagnosis and treatment of tuberculosis, and in tuberculosis reporting systems prior to the launch of the TDTC in 2003. Briefings were organised at the hospital level for other staff. However, nurses trained in tuberculosis care have been transferred out of both hospitals to other health facilities since 2008. Since 2004, the TDTC nurse and one laboratory technician per TDTC in each hospital have been supervised 3 to 4 times per year by the regional NTCP coordinators and once per year by a NTCP manager from the central level. The supervisions focus on reviewing tuberculosis data collection and reporting tools, and on assessing the implementation of tuberculosis control directives. However, the NTCP coordinators bypass the DHS to supervise and directly monitor tuberculosis care in the two DHs. Additionally, the nurses working in the outpatient departments in both hospitals and the specialised radiograph technician of the DHA have never been trained in tuberculosis diagnosis and treatment, nor have they been supervised by the NTCP.

Concerning the supervision of routine activities by the DHS, all health centre nurses and hospital managers in both health districts explained that they were primarily supervised during mass immunization campaigns by people coming from the district, regional, or central level or from international organisations.

At the health district A, a DHS staff member stated that ‘*we are not invited to supervise; the regional staff of the tuberculosis programme just inform us by phone when they arrive at the DH for supervision*’. Another member of the DHS explained that health centres were only supervised 2 to 3 times per year if they received support from programmes. Concerning the supervision of the hospital, this staff member added that ‘*we do not supervise the hospital because if we ask for data that could have a link with their financial revenue, they do not provide them to us’*.

In DHB, a laboratory technician stated that ‘*the supervisor teaches us how to process and read sputum smears, and I use this competency for other exams, such as blood smears’*. The TDTC nurse acknowledged that skills acquired in counselling were used to improve communication with non-tuberculosis patients. A member of the DHS noted that that*, ‘before 2008, the district medical officer was also the director of the hospital; however, since 2009, the tuberculosis supervisors merely inform the district medical officer and supervise independently*’. Long distances and competing priorities were given as the main reasons for the lack of supervision of health centres.

### Effects of the NTCP on the technical capacity of DHs

The NTCP allocated resources only to health facilities designated as TDTCs (see Table 7). These resources were meant to enable the hospitals to diagnose and treat tuberculosis even though some materials such as microscopes provided by the NTCP were also used for processing samples from the exams of patients without tuberculosis. The NTCP supplies first-line anti-tuberculosis drugs free of charge to patients, reagents for sputum smears, slides, and sputum collectors on a quarterly basis. However, the anti-tuberculosis drugs are frequently out-of-stock. During the data collection period, the Rifampicine-Isoniazide-Ethambutol-Pyrazinamide drug combination was out-of-stock for more than one week in DHA, and a hospital manager said that the regular provision of tuberculosis drugs by the NTCP is an important concern. Other drugs administered for the treatment of co-morbidities are to be purchased by patients. The two TDTCs were equipped with microscopes in 2003 and 2007. Since 2010, the NTCP quarterly allocates 15000 FCFA to each TDTC nurse to support some TDTC running costs.

In both hospitals, tuberculosis patients pay consultation fees (600 FCFA) and fees for sputum smear exams (1000 FCFA). In the DHA, chest radiography (5000 FCFA) and hospitalisation (3000 FCFA) fees are also paid by patients, while biopsies are performed free of charge in suspected extra-pulmonary tuberculosis cases.

At the regional level, one interviewee stated that the policy of the tuberculosis programme is to use the smear sputum fees to cover the operating costs of the TDTC. The two DHs receive approximately the same technical capacity support, while the DHA already has more resources than the DHB.

In 2006, the DHA received a motorcycle from the NTCP to trace tuberculosis patients who had been lost to follow up. A nurse stated that ‘*according to tuberculosis programme managers, this motorcycle should only be used for tuberculosis activities, but the hospital director also uses it for other hospital outreach activities, such as vaccination’*. The 30-bed tuberculosis ward was rehabilitated and equipped in 2008 using hospital revenues. The TDTC nurse collected and managed fees for sputum smears. He explained that he also gave incentives to the laboratory technician who processed sputum smears. Other staff members who identify suspected tuberculosis cases among outpatients or inpatients do not receive any incentives from the NTCP.

In the DHB, the NTCP rehabilitated a 4-bed building for the hospitalisation of tuberculosis patients in 2006. The TDTC nurse argued that sputum smear fees were still centralised and managed like other user fees collected in the hospital and complained of lack of office equipment.

### Effects of the NTCP on the routine HIS of DHs

In this section, we present the various factors (technical, organisational, and behavioural), as well as the processes pertaining to the tuberculosis HIS and how they affect the routine HIS in both DHs (see Table 8).

Technical factors

In both hospitals, the NTCP introduced standardised printed registers and tools for data collection and reporting on tuberculosis. There is one register for sputum smear results and another for diagnosed tuberculosis cases. There are also two quarterly reporting forms: one for tuberculosis cases detected during the previous trimester and one for the tuberculosis prognosis of cases detected 9 months earlier. The two TDTC nurses acknowledged that ‘*completing these registers is simple even if it takes a lot of time’*.

The NTCP has not modified the routine data collection and reporting tools. Registers for routine data collection are printed locally in DHA and manually designed in DHB, with contents varying between wards and over time. In the DHA, the monthly routine information tool designed by the Ministry of Public Health has not been available since 2004. Computers are available in both hospitals and DHS offices, but there is no specific software for managing HIS.

Other programmes, such as for malaria, HIV/AIDS, and immunisation, have their own data collection and/or reporting tools. There were neither standardised routine data collection registers for hospitals nor a standardised reporting tool that bundled data from specific programmes and general care.

Organisational factors

Each processed sputum smear is registered at the laboratory in both hospitals. Following the receipt of laboratory results, patients diagnosed with tuberculosis are registered by the TDTC nurse who each quarter manually collates and records data on the two reporting forms. The reports are directly sent to the regional coordinator by both TDTCs, bypassing the district level. During the TDTC supervision activities that we observed, all tuberculosis data collection tools and reports were checked for accuracy. There was, however, no supervision for the routine HIS. An annual evaluation meeting was organised at the regional level for tuberculosis control activities, but it was only attended by the TDTC nurses and hospital directors. This meeting focused on validating the annual quarterly tuberculosis reports of each TDTC and on planning for the year. Internet services are available in both hospitals and DHS offices, but no electronic transmission of data or provision of feedback is available.

In both hospitals, routine inpatient and outpatient data are inputted manually in specific registers. At the end of the month, the nurses in charge of a ward manually collate the data.

In the DHA, the person in charge of health statistics uses the ward data to complete the Excel spreadsheets designed for that purpose. Some indicators are automatically calculated and compared with previous periods. This report is sent each month to the Church’s Health Department.

No specific indicator related to hospital care is calculated for the DHB, as the nurses in charge of the wards send the data to the superintendent, who then fills them using the monthly HIS tool designed by the Ministry of Public Health.

Overall, the tuberculosis control HIS was designed in parallel with the routine HIS in both hospitals.

Behavioural factors

TDTC nurses are highly motivated and take care to properly complete their reports. They have progressively acquired skills in the collection, verification and collating of data on tuberculosis.

At the DHB, the TDTC nurse stated that ‘*tuberculosis reports are carefully checked to avoid errors, as the supervisors will review these reports and registers’*. He added that ‘*this attitude helps me to pay particular attention to my monthly report of the medicine ward, but the routine report is not checked*’. Additionally, a hospital manager argued that ‘*nobody cares if you complete or submit your general monthly report*’. A nurse in charge of a ward noted that ‘*other nurses are not motivated to complete the registers, and they consider reporting to be an additional task not related to their activities’.*

This situation was in line with what a nurse in DHA noticed; namely, ‘*there is too much work and too little time for filling in all the registers and doing reports’*. Also, the nurse in charge of the statistics in DHA who was recruited in 2008 noted that he has never seen the hospital routine reporting form designed by the Ministry of Public Health.

Routine information processes

In both hospitals, we observed that routine registers were not correctly completed and that there were missing data for some patients, such as their age, gender, and prognosis. From to 2003 to 2010, tuberculosis registers and reports were rigorously filled in, were well maintained, and had the supervisors’ recommendations written on them. The TDTC nurses compiled the tuberculosis data without further analysis. The role of the DH managers in running the HIS was limited to transmitting the data to the regional level; no local analysis of these data was performed (see Figure 2). Before 2003, the tuberculosis registers in the two DHs had lot of missing information such as the demographic characteristics of the patients, the type and the prognosis of the tuberculosis cases.

In DHA, routine reports designed by the Ministry of Public Health were only sent to the DHS from 1998 until 2004, but the hospital regularly sends reports to the Church’s Health Department. In DHB, only the 2010 monthly reports were found at the DHS level and lacked any analysis. At the regional level, only few routine monthly hospital reports were found for the entire region.

### Adaptive measures

Apart from some few differences, NTCP interventions were roughly similar in both DHs and do not depend on the scale of patients received, the institutional identity, the resources available, nor the management style. However, each DH adopted adaptive measures to ensure tuberculosis care. These adaptive measures were sometimes in contradiction with the NTCP guidelines such as systematic hospitalization of tuberculosis patients in DHA, or sputum smear fees not managed by the TDTC nurse in DHB.

At the DHA, a laboratory technician was dedicated to processing sputum smears while a nurse only took care of tuberculosis patients. This helped the hospital to deal with the high number of tuberculosis cases. During morning meetings, guidelines regarding tuberculosis detection were re-discussed with nurses working in the general outpatient department so as to improve their skills in tuberculosis detection and mitigate the lack of formal training and supervision specifically addressing tuberculosis care. The hospital invested from its revenues to rehabilitate and equip a 30-bed ward for hospitalizing tuberculosis patients. All tuberculosis patients were hospitalized as explained by the TDTC nurse ‘*to ensure the direct-observed treatment and improve their compliance to the treatment’.* The chest radiography was done to facilitate the diagnosis of pulmonary tuberculosis as well as biopsies for some extra-pulmonary tuberculosis. In 2010, the DHA diagnosed 49% and 25% of all regional extra-pulmonary tuberculosis and SNPT respectively while the health district A only shares 15% of the total regional population. But the DHA did not receive more support from the NTCP than the DHB. The DHB has a less number of staff and uses a nurse in charge of a ward as the TDTC nurse to take care of tuberculosis patients while its three laboratory technicians process sputum smear exams. The ward rehabilitated by the NTCP was out-of use and tuberculosis patients were not hospitalized.

### Missed opportunities for building synergies between the NTCP and the local health system

Several opportunities were missed by both the NTCP and the health systems managers to build synergies between the NTCP and local health systems (see Table 9). Regarding human resources, such missed opportunities include the allocation of additional staff and their continuous in-service training, and the organisation of joint supervision by regional NTCP coordinator –as well as other programmes’ coordinators- and district health teams. This joint supervision could improve the competence, confidence and authority of the latter to further supervise alone tuberculosis care and other health activities at the district level. The district health teams that are at a better position to identify health system weaknesses and/or disruptive effects of disease control organisation on general health care could discuss these issues with programmes’ coordinators during joint supervisions.

**Table 9:** Missed opportunities for building synergies between the NTCP and local health systems

**Domains**	**Missed opportunities**
**Human resources**	Recruitment of additional staff
	Identification of training needs for DHs and health centres staff members
	Organisation of in-service training with the support of regional disease control programmes managers
	Reinforcement of competencies of the district management teams in organizing, monitoring and evaluating tuberculosis control activities
	Implication of district management teams in the supervision of TDTC
**Health Information System (HIS)**	Standardisation of routine data collection tools for all DHs
	Elaboration of a unique data reporting tools that bundles routine and programmes’ data
	Development of software for managing health data at DH and DHS levels
	Utilisation of the electronic system for data transmission and feedback between DHs, DHS and regional programme coordinations
	Reinforcement of capacities of district management teams and DH staff in the management of HIS for decision-making
**Technical capacity**	Identification of hospital technical needs and allocation of resources on the basis of hospital needs
	Submission of health system strengthening proposals to the Global Fund against HIV/AIDS, tuberculosis and malaria
**Health service delivery**	Organization of the referral system
	Development of the collaboration between HIV/AIDS and tuberculosis care at facility level

On HIS, synergies could have been built by the regional level through the standardisation of routine data collection, and the development of a unique data reporting tool that bundles routine and all programmes data to reduce multiple data reporting tools and duplication. Moreover, the alignment, reinforcement and integration of programme monitoring and evaluation systems into routine HIS could improve the capacity of general health facilities to produce quality health data for *both* programme and general care in a timely manner.

Regarding the capacity building, the ‘health system strengthening window’ opened by the Global Fund to fight AIDS, Tuberculosis and Malaria were not seized to submit proposal for strengthening the health system [35].

Finally, no well-functioning referral system – with an exchange of relevant patient information back and forth between peripheral facilities and DHs - existed. Such a system would have the potential to improve timely care for severe patients needing more technical expertise at the hospital level, and enhance continuity of care for more simple cases at the primary health care level.

## Discussion

A limitation of this study pertains to the case study design with cases purposively selected and limited in number. However, at the regional level, the NTCP coordination applies the same strategy in all DHs in which a TDTC is nested. Also, at the national level, there is a standardization of the policy and guidelines of the NTCP for all 10 health regions [36]. The two cases selected reflect the two types –public and pnfp - of DHs in Cameroon. All public DHs have a roughly similar managerial organization. Even though there will always be *some* differences between DHs, lessons have been learned on how to possibly improve the interface between the NTCP and DHs in Cameroon and in similar contexts. Caution is obviously due in any attempt to make broad generalisations on the basis of two cases studies conducted in a same region. Furthermore, some issues raised by this study could be further investigated using a more representative sample of districts or TDTCs for quantitative analysis.

Since 2003, in the Adamaoua region, the NTCP improved the ability of the DHs designated as TDTC to detect and treat tuberculosis cases by reinforcing the competency of staff, allocating equipment, reagents and anti-tuberculosis drugs free of charge, and putting in place a standardized HIS for tuberculosis control. The increase in the number of TDTCs has proven to be an effective strategy for improving the detection of tuberculosis cases and care for these patients [22].

Interventions by the NTCP do not appear to have strengthened local health systems in the manner required to achieve programme objectives and health system goals. Chee and colleagues [37] have made the useful distinction between ‘*health system support’* and ‘*health system strengthening’* activities. They classified the provision of inputs (equipment, drugs, and reagents) and trainings that are narrowly focused on specific disease control activities as ‘health system support’. Health system strengthening is ‘*accomplished by more comprehensive changes to policies and regulations, organizational structures, and relationships across the health system building blocks that motivate changes in behaviour and allow more effective use of resources to improve multiple health services’*[37]. This concept is similar to what Potter and Brough [38] classified as *sub-optimal* and *more effective* approaches to capacity building. The NTCP interventions consist mainly of ‘*health system support’,* and focus on tuberculosis control support that falls short in terms of addressing structural health system constraints and building strong and sustainable local health systems [39].

NTCP interventions in DHs were either continuous (e.g., through the procurement of anti-tuberculosis drugs), intermittent (e.g., through supervision and training) or sporadic (e.g., through the provision of laboratory equipment). Although most sub-Saharan health services are under-staffed and under-equipped [40], NTCP support in Cameroon remains largely limited to facilities identified as TDTCs, most of which are located in the DHs. The NTCP continues to under-perform, with drugs and reagents frequently out-of-stock, supervision taking place irregularly, and only one annual evaluation meeting being held. The annual evaluation meeting focuses mainly on validating programme outputs produced by TDTCs rather than on analysing and addressing constraints in tuberculosis care at the district level. Hospital capacity building focuses on procuring equipment and drugs for tuberculosis control and on training only one specialised nurse and one laboratory technician for tuberculosis care. In this way, a parallel system for tuberculosis care has emerged with specific staff and a specific HIS, with monitoring and supervision of tuberculosis control activities by the NTCP managers that bypass the DHS. There is little evidence that the NTCP actually improves general health care delivery or that it strengthens the functioning of either hospital. Additionally, the DHS teams in both settings are not involved in planning, monitoring, supervising or evaluating tuberculosis control activities.

With a few exceptions—namely, a motorbike allocated to the DHA and a rehabilitated building in DHB—the effects of the NTCP are similar in both DHs, despite differences between the two hospitals in terms of technical capacity, staff number, management, revenues, and general care performance. DHs attempt to adapt to ensure the functioning of the TDTC without negatively affecting the delivery of general care and to minimize any disruptive effects. This adaptive capacity seems particularly developed in DHA that has more resources, and a decentralized management style.

NTCP strengthening district health systems is not obvious in a context where the programme itself faces difficulties in funding its proper activities and in achieving its own objectives. Strengthening district health systems, however, does not always require extra resources – it also is a matter of well-thought and well-coordinated policies and management procedures. Notwithstanding this, including the health system strengthening dimension in the NTCP portfolio may contribute to go beyond specific programmatic activities. The NTCP could then search for additional funding and expertise to reinforce the general health system – for instance via the health systems strengthening component of the Global Fund to fight HIV/AIDS, Tuberculosis, and Malaria. Indeed, strong health systems are required if disease control programmes objectives are to be reached and sustained [41].

Hospitals that function well with regard to their three dimensions (spatial, managerial, and technical) also have better tuberculosis control outputs. The DHA, like other faith-based hospitals, has a good reputation, is reasonably well equipped, has more-committed and specialized staff in tuberculosis control and is led by a strong management team. The DHA possessed more revenues coming from multiple sources that can be used without strict hierarchical directives, and has invested in tuberculosis control as well as in other hospital activities independently of the NTCP’s support. Even though there was a reduction in outpatients, the number of inpatients remained stable indicating that simple cases were seen at the primary health care level leading to a better use of the hospital expertise for severe cases including tuberculosis cases.

On the contrary, the DHB is less equipped and has limited amenities for patient comfort. The staff are also less engaged and are managed at the central level, with a lower capacity to generate additional resources and less flexibility in the use of these resources. At best, the DHB has detected 50% of the expected SPPT cases. This could also be explained by the lower rate of referred patients from primary health care services, in comparison with DHA. Since 2006, the increase in number of staff and the allocation of new equipment to DHB have, however, led to improved hospital utilisation and to increased detection of tuberculosis cases. But other performance indicators remained sub-optimal. These findings support the hypothesis that enhancing the functioning of DHs leads to better outputs in terms of disease control. Therefore, it is in the NTCP’s interest that district health systems, including the DHs, function better.

Our study shows that the NTCP scarcely involves the DHS and that the DHS does not properly execute its role in planning, monitoring, supervising and evaluating all district health activities, including tuberculosis control. This situation reflects what Biesma and colleagues [8] have called ‘*missed opportunities*’, or opportunities not seized that can produce positive synergies between disease control programmes and the general health system [42,43]. These synergies are pre-requisites for attaining programme objectives and health system goals [44,45], and achieving long-term outcomes [41,44]. Therefore, a routine monitoring of the interface by managers of general health services and of programmes will provide avenues for optimizing the interaction between programmes and general health services by proactively searching and seizing all opportunities [46].

Currently, the strategy of the NTCP is a centralized strategy –with diagnosis and treatment of tuberculosis mainly in DHs-, using polyvalent staff in general health services. However, in 2011, the tuberculosis prevalence remained high −299 cases per 100000 inhabitants [47]. Therefore, case detection and follow up should be improved to achieve the NTCP objectives. We recommend a strengthening of existing TDTCs to deliver quality tuberculosis control activities, the implication of first line health services in the detection and referral of suspect tuberculosis patients to TDTCs, and finally a gradual capacitation of the first line health services for a progressive and genuine decentralisation of diagnosis, treatment and follow up of tuberculosis cases. This decentralisation is not antinomic of keeping a strong role for DHs in tuberculosis control.

## Conclusion

Well-managed DHs perform better in terms of tuberculosis control compared with those that are not well-managed. It is, therefore, in the interest of the NTCP and other programmes to develop and collaborate with strong local health systems and DHs that effectively fulfil their roles. Our analysis of the effects of the NTCP on human resources, the HIS and the technical capacity of DHs indicates that the tuberculosis programme *supports,* rather than *strengthens,* the local health system. There is potential for the NTCP to have a more effective role. Positive synergies between the NTCP and district health systems could be achieved if opportunities to strengthen the district health systems are seized. The question remains as to *why* managers do not take advantage of these opportunities. We recommend further analysis of this issue in the specific context of Cameroon.

## Endnotes

^a^1 US dollar equals 450 CFA francs.

## Abbreviations

DH: District Hospital; DHA: District Hospital A; DHB: District Hospital B; DHS: District Health Service; HIS: Health Information System; NTCP: National Tuberculosis Control Programme; PNFP: Private-Not-For-Profit; SPPT: Smear Positive Pulmonary Tuberculosis; TDTC: Tuberculosis Diagnosis and Treatment Centre.

## Competing interests

The authors declare that they have no competing interests.

## Authors’ contributions

BK conceived and designed the study. JMa, AB, JMe and BC revised and approved the study design. BK collected and analysed data, and drafted the manuscript. JMa, AB, JMe and BC critically revised the manuscript and have given final approval of the version to be published. All authors read and approved the final manuscript.

## Pre-publication history

The pre-publication history for this paper can be accessed here:

http://www.biomedcentral.com/1471-2458/13/265/prepub
